# Twelve-month effect of chronic pain self-management intervention delivered in an easily accessible primary healthcare service - a randomised controlled trial

**DOI:** 10.1186/s12913-018-3843-x

**Published:** 2018-12-29

**Authors:** Torunn Hatlen Nøst, Aslak Steinsbekk, Ola Bratås, Kjersti Grønning

**Affiliations:** 1 Department of Public Health and Nursing, Norwegian University of Science and Technology, Postbox 8905, 7491 Trondheim, Norway; 2 Center for Health Promotion Research, Norwegian University of Science and Technology, Trondheim, Norway

**Keywords:** Chronic pain, Long-term effect, Patient activation, Primary health care, Self-management

## Abstract

**Background:**

To investigate the effects after twelve months related to patient activation and a range of secondary outcomes on persons with chronic pain of a chronic pain self-management course compared to a low-impact outdoor physical activity, delivered in an easily accessible healthcare service in public primary care.

**Methods:**

An open, pragmatic, parallel group randomised controlled trial was conducted. The intervention group was offered a group-based chronic pain self-management course with 2.5-h weekly sessions for a period of six weeks comprising education that included cognitive and behavioural strategies for pain management, movement exercises, group discussions and sharing of experiences among participants. The control group was offered a drop-in, low-impact, outdoor physical activity in groups in one-hour weekly sessions that included walking and simple strength exercises for a period of six weeks. The primary outcome was patient activation assessed using the Patient Activation Measure (PAM-13). Secondary outcomes included assessments of pain, anxiety and depression, pain self-efficacy, sense of coherence, health-related quality of life, well-being and the 30-s Chair to Stand Test. Analyses were performed using a linear mixed model.

**Results:**

After twelve months, there were no statistically significant differences between the intervention group (*n* = 60) and the control group (*n* = 61) for the primary or the secondary outcomes. The estimated mean difference between the groups for the primary outcome PAM was 4.0 (CI 95% -0.6 to 8.6, *p* = 0.085). Within both of the groups, there were statistically significant improvements in pain experienced during the previous week, the global self-rated health measure and the 30-s Chair to Stand Test.

**Conclusions:**

No long-term effect of the chronic pain self-management course was found in comparison with a low-impact physical activity intervention for the primary outcome patient activation or for any secondary outcome.

**Trial registration:**

ClinicalTrials.gov: NCT02531282. Registered on August 212,015

## Background

Nearly one in five individuals (19%) in the adult European population have chronic non-cancer pain [1, 2], and certain countries such as Norway, have an even higher prevalence (30%) [1, 3]. Chronic pain is usually referred to as persistent pain lasting for three months or more [4] with a majority of individuals reporting symptoms beyond one year [1, 5]. Chronic pain is characterised by extensive and fluctuating symptoms [5, 6] with a broad impact on quality of life [5], and thus it requires a range of strategies for self-management [7]. Self-management refers to one’s ability to manage the chronic condition and its treatment, to adopt to physical and psychological changes and to adhere to lifestyle modifications [8]. For chronic pain, self-management strategies often refers to methods a person uses to limit the impact of pain on everyday life, moods and functions, both at home and work [9]. This typically includes actions such as physical activity [10], activity pacing [11], and a focus on how to use one’s mind to manage pain [12].

As a crucial element to reducing the impact of chronic pain, at both the individual and the population levels, affected individuals must play a central role in the management of the pain and its associated consequences [9]. This includes both self-management activities performed by the individuals and healthcare services that aim to support patients apply self-management strategies [9, 13]. Interventions that support self-management emphasise the process of central self-management skills, such as self-efficacy development, self-monitoring, goal-setting and action planning, decision-making, problem-solving, self-tailoring and partnerships between the views of patients and health professionals [14]. Patient activation, which includes the knowledge, skills and confidence people have to manage their health [15], is a concept closely connected to self-management initiatives because self-management requires people to be empowered and to possess the necessary information, resources and skills to make decisions and to manage their health on a day-to-day basis [16]. Typically, the aim of self-management interventions is thus to empower people to be active partners in healthcare by providing information and skills to enhance the ability to self-manage health [17].

The processes of adopting to new self-care activities and developing self-management skills are likely to require time [18, 19]. The ability to self-manage can be described as a continuum, with individuals exhibiting varying levels of the ability [19]. To change perceptions towards pain, it has been emphasised that new strategies must be practiced and preferably result in positive experiences [19]. Moreover, it often requires continuing efforts to maintain the learned behaviours and strategies over time [20]. Consequently, there is a need for knowledge related to pain self-management interventions’ effects over time.

To some degree, this has previously been investigated. In one review of multidisciplinary biopsychosocial rehabilitation for chronic low back pain, small long-term improvements in pain and disability were observed [21]. Another review of osteoarthritis, for which pain is a common symptom, showed small benefits in terms of self-management skills, pain osteoarthritis symptoms and functions up to 21 months, although the clinical importance of these benefits was unclear [22]. In addition, a review regarding chronic musculoskeletal pain showed minor or statistically insignificant differences after eight months of group-delivered self-management courses [23]. A recent review reached a similar conclusion based on that self-management interventions were found to have marginal benefits on self-efficacy, pain intensity, physical function and physical activity for patients with chronic musculoskeletal pain [20]. Thus, there is some evidence for the long-term benefits of chronic pain self-management interventions, although the evidence is inconsistent [20, 24]. Accordingly, there is still a need for more research on the long-term effects of chronic pain self-management interventions [25], especially for chronic pain self-management interventions delivered in non-specialist settings to which people can self-refer, regardless of a specific diagnosis.

Because changing perceptions and behaviours towards pain most likely require time, simultaneously to that newly learned behaviours may be difficult to maintain [20], the effects of interventions should be investigated over different time spans. The authors have previously investigated the effect after three months of a group-based chronic pain self-management course delivered by an easily accessible healthcare service in primary care [26]. The investigations did not reveal any statistically significant differences in favour of the self-management course compared to a low-impact physical group activity. Because a period of three months may be too short for participants to benefit from an intervention, the assessments of the effects of the self-management course should also include a more long-term perspective.

The aim of this study was therefore to investigate the effects after twelve months related to patient activation and a range of secondary outcomes on persons with chronic pain of a group-based chronic pain self-management course compared to a low-impact outdoor physical activity delivered in an easily accessible healthcare service in public primary care.

## Methods

This was an open, pragmatic, parallel group, randomised controlled trial (RCT) conducted from August 2015 to December 2017. The protocol for the trial [27] has been published previously. There were no changes to the methods described in the protocol after the trial’s commencement.

### Ethics

The participants were informed about the trial both orally and in writing, and a written consent to participate was collected before enrolment. The Regional Committee for Medical and Health Research Ethics in South East Norway approved this study (2015/ 1030/ REK sørøst). The trial was registered at Clinical Trials.gov in August 2015 (NCT02531282).

### Setting

The trial setting was a Healthy Life Centre (HLC) in a major city in central Norway that serves a population of approximately 190,000 inhabitants. The HLCs are part of Norwegian public primary healthcare services and aim to reach persons of all ages at risk of developing, or those who already have developed, a non-communicable disease and require help to change health behaviours and to manage health challenges [28]. People can attend HLC activities with or without a referral [29]. In line with the general self-management initiatives increasingly shifting from specialised healthcare services to primary healthcare services in Norway [30], the HLCs are gradually incorporating self-management initiatives as part of their services.

### Participants

Participants could both be referred to by healthcare practitioners or self-refer in to the trial. The trial inclusion criteria were adults 18 years or older, with self-reported pain for three months or more who were able to take part in group discussions in Norwegian. In addition, they agreed to accept randomisation to one of the trial interventions after a full explanation of the trial. The exclusion criteria included an inability to participate in a low-impact physical activity for one hour, pain arising from malignant diseases, and lacking the capacity to consent.

Recruitment for the trial was communicated through posters and information leaflets distributed to general practitioners, physiotherapists, relevant departments at the hospital, Norwegian Labour and Welfare Administration offices and other relevant organisations in the municipality. Advertisements were placed in local newspapers, websites, social media and email invitations to patient organisations. Those interested in participating were encouraged to contact the first author by either phone or email. The first author checked the eligibility criteria, provided additional information about the trial and scheduled appointments for baseline assessments.

### Procedure, randomisation and blinding

Following the baseline assessments, the participants were consecutively, individually and randomly allocated to one of the two trial arms using a computer-based Internet trial service provided by a third party (Unit for Applied Clinical Research at the Norwegian University of Science and Technology, NTNU). A 1:1 ratio and a stratification for gender were used. Those involved in the trial were blinded to the block sizes.

Immediately after the randomisation, the participants were informed of their allocation by either phone or by an email from the first author. The research assistant who conducted the physical ability test at the follow-up appointments was blinded to allocations; otherwise, it was an open study, including the outcome assessments.

The outcomes were assessed at the baseline, and at three, six and twelve months after the completion of the intervention. The assessments after six and twelve months are reported here. At the baseline, the self-administrated questionnaire was completed with the first author available for questions. For the follow-up appointments, the questionnaires were collected when the participants met for the 30-s Chair to Stand Test.

### Description of the interventions

Both the self-management course and the low-impact, outdoor physical activity were developed by the HLC staff. There was no user fee for participation or any other financial support offered to the participants in either of the groups.

### The self-management course

The chronic pain self-management course, developed locally by the HLC staff in cooperation with a patient organisation representative, aimed to increase the participants’ knowledge, skills and confidence in managing everyday life with chronic pain [27]. The course was developed in accordance with the characteristics of self-management interventions [14], recommendations in the literature (e.g., [31–35]), the guidelines of the HLC [28] and personal experiences working with behavioural changes and the self-management of chronic conditions. Hence, the course addressed central self-management skills such as goal setting, action planning, and problem solving, and focussed on empowering the participants to play an active role in their healthcare. The chronic pain self-management course included education introducing cognitive and behavioural strategies for pain management [31–33, 35], pain theory, discussions of barriers in everyday life due to chronic pain, and techniques to deal with fatigue, poor sleep, frustration and isolation. For the movement exercises concluding each session, principles from psychomotor physiotherapy were applied [34]. The purpose of the exercises was to improve balance, posture and breathing, and to provide participants with techniques to increase body awareness and their ability to relax. In addition, the course emphasised group discussions and sharing of experiences among participants.

The self-management course was delivered as a weekly 2.5-h group session during the daytime (12.30 pm- 15.00 pm) for six weeks, for a total of 15 h. Two dedicated employees with professional backgrounds as physiotherapists experienced in working with behavioural changes, self-management and chronic pain facilitated the self-management course. One of the physiotherapists involved in developing and delivering the course was educated within psychomotor physiotherapy and had extensive experience from a multidisciplinary hospital pain clinic.

The guidelines regarding how to carry out the self-management course are available through the published protocol [27].

### The control group activity

The low-impact physical outdoor activity offered to the control group was an existing activity at the HLC. This activity was chosen because it offered a group activity with an opportunity to meet others with similar health challenges and because physical activity has shown beneficial effects on chronic pain conditions [36–38]. The low-impact outdoor physical activity was delivered as a weekly one-hour, drop-in session during the daytime (13.00 pm - 14.00 pm) for six weeks for a total of six hours. Two instructors familiar with physical exercise led the activity, which consisted of walking and simple strength exercises (e.g., squats and push-ups against a tree or a bench). A popular hiking trail was used for the activity. The participation was voluntarily, which is in line with the drop-in policy for this type of activity at the HLC. There was no education presented to the control group.

### Outcome measures

Participants’ characteristics, such as gender, age, marital status, education, employment status, main reason for pain categorised according to the International Classification of Primary care-2 (ICPC-2) and whether the individual suffered more than two chronic conditions were collected at the baseline assessment. At the follow-ups, the participants were asked whether there were any changes to these characteristics and about their healthcare utilisation during the previous three months, i.e., the number of visits to general practitioners, physiotherapists and hospitals or rehabilitation centres.

### Primary outcome

The chronic pain self-management course was hypothesised to strengthen the participants’ engagement in and knowledge of available health resources, which consequently was expected to lead to a higher level of patient activation. Thus, patient activation was chosen as the main outcome [27], and was measured using the Patient Activation Measure, PAM-13 [39]. The PAM-13 contains 13 statements to which the participants indicate their level of agreement on a four-point Likert scale, from 1 = ‘strongly disagree’ to 4 = ‘strongly agree’ with an additional ‘not applicable’ option. The raw score is transformed to a total score ranging from 0 to 100 [40], with higher scores indicating that the individual is more activated to adopt and to maintain healthy behaviours and self-management strategies for their illness, even under stress [15]. When participants answered that a statement was not applicable to them, the data was treated as missing. A total score was generated if participants answered at least 10 of the 13 statements [40].

The PAM-13 scores can be divided into four levels of activation [39]. Level 1 (score 0.0–47.0) indicates that a person may not yet understand that the patient’s role is important. Level 2 (score 47.1–55.1) indicates a lack of confidence and knowledge to take action. Level 3 (score 55.2–72.4) indicates that a person is beginning to engage in recommended health behaviours, whereas level 4 (score 72.5–100.0) indicates that a person is proactive regarding their health and engages in several recommended health behaviours [41, 42]. Patient activation levels have been used in studies as cut-off values to stratify participants and to investigate the effects of interventions in accordance with the different levels [41, 43, 44].

The PAM-13 is considered useful for assessing patient engagement in the management of chronic illness, including chronic pain, and for assessing sensitivity to changes in several groups and populations [15, 39, 45]. The measure has been translated into Norwegian (Cronbach’s alpha 0.91) [46]. Studies have shown that the Norwegian version of the measure is valid and reliable when tested for patient education interventions in a Norwegian hospital [46], in a RCT of hospital out-patient self-management education for patients with polyarthritis (Cronbach’s alpha 0.80) [47] and in a RCT of mental health treatment (Cronbach’s alpha = 0.87) [43]. Relevant for this study, the PAM-13 has been used in the above-mentioned study on polyarthritis patient education [47] and in an evaluation study of self-management interventions, including persons with chronic pain [45]. In the current study, the Cronbach’s alpha at the baseline was 0.75.

### Secondary outcomes

Several secondary outcomes were chosen in consideration of the recommendations from the Initiative on Methods, Measurement and Pain Assessment in Clinical Trials (IMMPACT) [48, 49], systematic reviews on self-management [17, 23, 24, 50] and findings from studies on persons with chronic pain and self-management (e.g., [47, 51, 52]).

Experiencing chronic pain was the main inclusion criteria. Therefore, pain severity and pain interference were assessed using the Brief Pain Inventory (BPI) [53].The instrument includes four questions related to severity and seven questions regarding interference where all items are rated on 0–10 scales, with 10 being pain as bad as one can imagine, or pain that interferes completely. In addition, the instrument includes one item that asks about the percentage of pain relief with the use of analgesics [53]. The instrument has been translated to Norwegian (Cronbach’s alpha 0.87 for pain severity and 0.92 for the interference scale) [54] and has been used in Norwegian studies of a multidisciplinary pain management programme [55] and among patients with osteoarthritis (Cronbach’s alpha > 0.80) [56]. In the current study, the Cronbach’s alpha at the baseline was 0.81 for pain severity and 0.86 for pain interference.

The experience of pain during the previous week was assessed using a one-item 100 mm Visual Analogue Scale (VAS) [57]. The participants were asked to draw a vertical mark on the 100 mm line indicating their average pain during the previous week. The scale’s anchoring points were no pain (0) and intolerable pain (100). The VAS has been validated and found to be reliable in the assessment of chronic pain [57].

Psychological distress is commonly reported among individuals suffering chronic pain [2, 5], which makes this a relevant domain to assess. Anxiety and depression were assessed using the Hospital Anxiety and Depression Scale (HADS), which consists of 14 items divided into two subscales, with seven items each for depression and anxiety [58]. Each item is rated from not experiencing symptoms (0) to experiencing symptoms nearly all the time (3). This instrument has shown good validity and reliability for patients with musculoskeletal pain (Cronbach’s alpha for the anxiety subscale 0.83 and for the depression subscale 0.84) [59] as well as in a Norwegian large population study (The Nord-Trøndelag Health Study, HUNT) (Cronbach’s alpha 0.80 for the anxiety subscale and 0.76 for the depression subscale) [60]. In the current study, the Cronbach’s alpha at the baseline was 0.73 for the depression subscale and 0.76 for the anxiety subscale.

Self-efficacy concerns the confidence people have that they can successfully execute a course of action to accomplish a desired outcome in a given situation [61]. This was measured using the Pain Self-Efficacy Questionnaire (PSEQ), which specifically assesses beliefs regarding one’s ability to accomplish various activities, despite the pain [62]. The PSEQ includes 10 items that respondents rate on a scale from 0 to 6 regarding how confident they are that they can perform an activity at present despite pain, where 6 equals completely confident [62]. The questionnaire has been tested in large samples of heterogeneous patients with chronic pain (Cronbach’s alpha 0.92) [62] and has been translated and validated in several languages and populations [63]. It has previously been translated for use in a Norwegian study (Cronbach’s alpha not reported) [64]. In the current study, the Cronbach’s alpha at the baseline was 0.84.

A sense of coherence has been suggested to be an important coping mechanism and strategy for people with chronic musculoskeletal pain [65] and is related to salutogenesis, which is fundamental to the activities at the HLC [28]. Therefore, this was included as an aspect to assess by using the Sense of Coherence (SOC) scale [66]. The 13 items of the scale measure the perception of the environments’ comprehensibility, manageability and meaningfulness, with each item scored using a range from 1 to 7. The score of each item is summed to a total score, with a range from 13 to 91. The higher the score, the stronger the sense of coherence. The SOC scale has been found to be a reliable, valid and cross-culturally applicable instrument that measures how people manage stressful situations and stay well (Cronbach’s alpha in 127 studies 0.70–0.92) [66]. The Norwegian version of the SOC-13 has been used in a study of patients with long-term musculoskeletal pain (Cronbach’s alpha not reported) [67] and in a study on multidisciplinary rehabilitation for patients with chronic musculoskeletal pain (Cronbach’s alpha 0.83) [68]. In the current study, the Cronbach’s alpha at the baseline was 0.87.

Living with chronic pain often affects people’s health-related quality of life [69]. The generic health-related quality of life was assessed using the EuroQoL (EQ-5D-5 L) [70]. This instrument provides five levels to answer each of the dimensions mobility, self-care, usual activities, pain/ discomfort, and anxiety/ depression [71]. The descriptive score was converted to an index value of health status using the Danish value set, giving a range from 1 (perfect health) to 0 (death) [70, 71]. The instrument has been validated in similar populations [72], as well as in a Norwegian context (Cronbach’s alpha 0.69) [73]. In the current study, the Cronbach’s alpha at the baseline was 0.55.

In addition to the assessment of health-related quality of life, the participants’ experiences related to global well-being during the previous month was assessed using the Arizona Integrative Outcomes Scale (AIOS) by means of a one-item 100 mm long VAS [74]. The question asked was ‘Reflect on your sense of well-being during the last month. Take into account your physical, mental, emotional, social and spiritual condition, and mark the line for your summarised overall sense of wellbeing’. The scale’s anchoring points were ‘worst you have ever been’ (0) and ‘best you have ever been’ (100) [74]. The AIOS has been found to be a valid measure for assessing well-being [74], and it has previously been used in a Norwegian study including persons that experience chronic pain [47].

In addition, the participants’ global self-rated health was assessed using the question ‘By and large, would you say that your health is:’ followed by the options ‘poor’, ‘not so good, ‘good’, ‘very good’ and ‘excellent’. This question is similar to a question asked in a major population study in Norway (HUNT) [75].

Because chronic pain can affect physical functioning and physical exercise has been shown to have beneficial effects on chronic pain [36, 37], two questions were included related to physical functioning. First, physical activity was assessed based on the average number of times participants exercised per week using the question: ‘How often do you exercise on average? (exercise refers to walking, skiing, swimming and working out/ sports)’ followed by the options ‘never’, ‘less than once a week’, ‘once a week’, ‘2-3 times a week’ and ‘nearly every day’. This question was used in a major population study in Norway [75] and in investigations of associations between exercise and chronic pain [76]. Second, as an objective measure of physical ability, the 30-s Chair to Stand Test was used to measure lower body strength [77]. Participants were told to sit on a chair with their hands on the opposite shoulder and feet flat on the floor. On a signal, they rose to a full stand and returned to a fully seated position, without using their arms. The score is the total number of unassisted stands during the 30-s time frame. The measure has shown to be valid and have good test-retest reliability in older adults [77] and has been used in studies including patients with fibromyalgia [78], knee and hip pain [79] and arthritis [80].

### Sample size

The findings of a RCT investigating the effect of a patient education programme for patients with polyarthritis in which the PAM was one of the secondary outcomes was applied to calculate the sample size [47]. Thus, the sample size was calculated to detect a clinically important difference, defined as six points on the PAM-13 from the baseline to the 12-month follow-up. A linear mixed model was used assuming the correlation among the participants to be 0.5, with a standard deviation (SD) of 13 [47]. The significance level was set to 5%, and the power was set to 80%, which yielded a number of 55 participants for each trial arm. Allowing five dropouts in each trial arm, the aim was to recruit 120 participants.

### Statistics

All the outcome measures were found to be approximately normally distributed. The confidence level was set at 95%, and the predefined cut-off level for statistical significance was set at *p* ≤ 0.05. No interim analysis was performed.

The effect of the intervention was assessed using intention to treat (ITT) and per-protocol (PP) analyses. The PP criterion was that participants had been present for a minimum of three of the six sessions. The PP analyses provided similar findings and did not change any conclusions regarding the interventions. Thus, they are not further discussed.

The between group differences are analysed only after 12 months, and the within-group changes after six and 12 months.

Analyses of the primary and secondary outcomes were performed using a linear mixed model. The participants’ identification number (ID) was specified as a random effect to allow participants to begin at different levels of the outcome in question. The effects of intervention and time were specified as fixed with the following values: 1) ‘baseline’, 2) ‘control 6 months’, 3) ‘intervention 6 months’, 4) ‘control 12 months’ and 5) ‘intervention 12 months’, acknowledging that differences between groups at the baseline were due to chance. The missing data were managed using the mixed linear model in which all available data are used. The regression assumptions were checked [81], resulting in satisfactory values. The analyses of the estimated changes from the baseline to six months and from the baseline to 12 months were performed separately.

Changes in work status and pain medication (categorical data) since the last assessment were analysed using Pearson’s Chi-Square test or Fisher’s exact test. The frequency of healthcare utilisation during the previous three months was compared between the groups using t tests.

One exploratory post-hoc subgroup analysis was performed to investigate whether changes in the primary outcome (PAM-13) varied according to patient activation levels at baseline. The reason for performing this analysis was a discussion after the study began regarding which groups of participants the course could possibly be best suited for, partly due to considerations made after a qualitative study about expectations towards participation with a selection of the participants in the RCT [82]. Because there were few participants at the lowest patient activation levels, patient activation levels 1 and 2 were combined, creating three subgroups. Distribution according to the different PAM-levels at baseline is displayed in Table 1. A linear regression analysis was performed to test for an interaction between the baseline patient activation level and allocation. The dependent variable was the change in PAM-13 from the baseline to twelve months. The independent variables were the PAM-13 level at the baseline and allocation (intervention or control group).

**Table 1:** Baseline characteristics of participants

Characteristics	ALL (*N* = 121)	INTV (*n* = 60)	CTRL (*n* = 61)
Female, n (%)	106 (87.6%)	53 (88.3%)	53 (86.9%)
Age years, mean (SD),	52.7 (11.7)	52.1 (11.4)	53.3 (12.1)
(range)	(23–74)	(27–71)	(23–74)
Living with someone, n (%)	86 (71.1%)	43 (71.7%)	43 (70.5%)
Highest level of education, n (%)			
lower secondary school or less	8 (6.6%)	4 (6.7%)	4 (6.6%)
upper secondary school	56 (46.3%)	28 (46.7%)	28 (45.9%)
higher education (college or university)	57 (47.1%)	28 (46.7%)	29 (47.5%)
Main reason for pain, n (%):			
musculoskeletal diseases, ICPC-2 chapter L	93 (76.9%)	46 (76.7%)	47 (77.0%)
neuro system diseases, ICPC-2 chapter N	16 (13.2%)	10 (16.7%)	6 (9.8%)
general and unspecified, ICPC-2 chapter A	12 (9.9%)	4 (6.7%)	8 (13.1%)
Pain duration, n (%)			
7–11 months	2 (1.7%)	2 (3.3%)	0 (0%)
1–5 years	24 (19.8%)	12 (20.0%)	12 (19.7%)
6–9 years	19 (15.7%)	11 (18.3%)	8 (13.1%)
10 years or more	76 (62.8%)	35 (58.3%)	41 (67.2%)
More than one chronic condition, n (%)	76 (62.8%)	32 (53.3%)	44 (72.1%)
Work status, n (%)			
working, full or part time	31 (25.6%)	13 (21.7%)	18 (29.5%)
disability pension, full or graded	56 (46.3%)	33 (55%)	23 (37.7%)
sick leave, full or graded	20 (16.5%)	8 (13.3%)	12 (19.7%)
retired	14 (11.6%)	6 (10.0%)	8 (13.1%)
Pain medication, n (%):			
prescription-only	51 (42.1%)	23 (38.3%)	28 (45.9%)
without prescription	41 (33.9%)	19 (31.7%)	22 (36.1%)
do not use pain medication	29 (24.0%)	18 (30.0%)	11 (18.0%)
Healthcare utilization, last 3 months:			
visits general practitioner, mean (SD)	1.9 (1.9)	1.6 (1.7)	2.1 (2.0)
visits physiotherapist, mean (SD)	4.8 (6.3)	4.5 (5.9)	5.1 (6.8)
stays rehabilitation centre, mean (SD)	0.07 (0.3)	0.1 (0.3)	0.05 (0.2)
visits hospital outpatient clinic, mean (SD)	0.6 (1.1)	0.5 (0.9)	0.6 (1.3)
admission hospital, mean (SD)	0.1 (0.7)	0.2 (1.0)	0.02 (0.1)
number of days, mean (SD), (range)	0.1 (0.8) (0–8)	0.2 (1.2) (0–8)	0.02 (0.1) (0–1)
PAM-13 level at baseline	*N* = 119	*n* = 58	*n* = 61
Level 1	16 (13.4%)	9 (15.5%)	7 (11.5%)
Level 2	12 (10.1%)	3 (5.2%)	9 (14.8%)
Level 3	61 (51.3%)	32 (55.2%)	29 (47.5%)
Level 4	30 (25.2%)	14 (24.1%)	16 (26.2%)

*INTV*: intervention group; *CTRL*: control group; *ICPC- 2*: International Classification of Primary Care, Second edition; *PAM-13*: Patient Activation Measure 13

The first author performed the analyses, which were overseen and discussed with the co-authors and a statistician. All the analyses were performed using Stata Statistical Software (Release 14; StataCorp LP, 2014, College Station, TX, USA).

## Results

### Participant flow

**Fig. 1:**
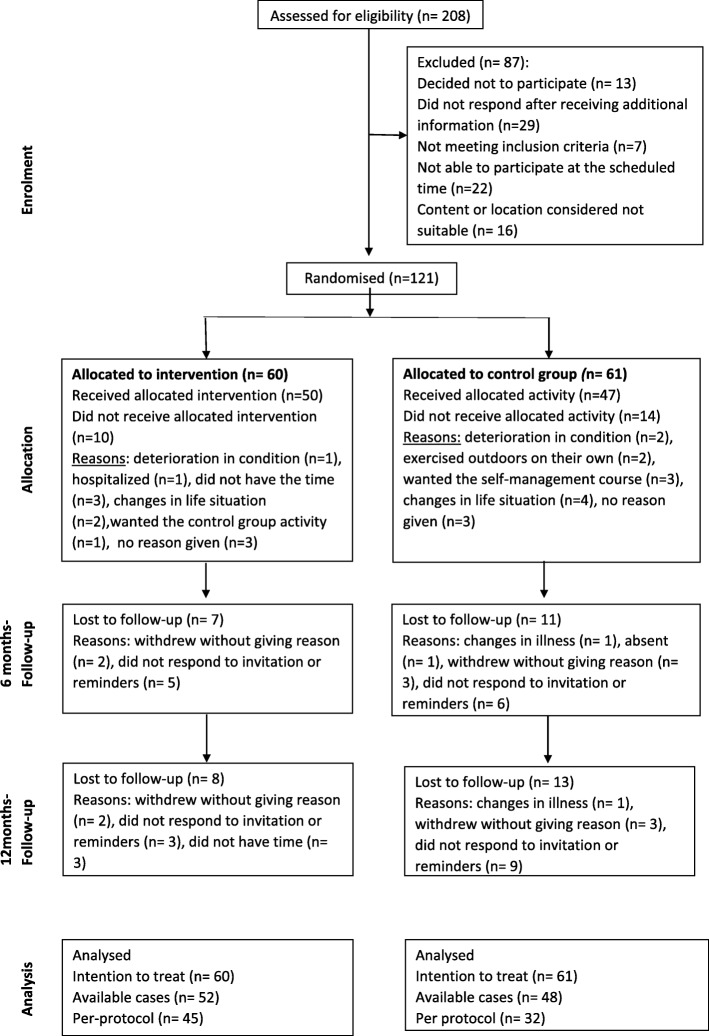
Participants flow through the study

The flow of the participants through the trial is shown in Fig. 1. Of the 208 people contacting the trial, 121 participants were included and randomised to either the chronic pain self-management course group (*n* = 60) or the low-impact physical activity group (*n* = 61). The number of participants who answered the questionnaires at the follow-ups were equally distributed to the trial arms.

### Baseline characteristics

The groups were comparable at the baseline. They consisted of mostly women (88%), the mean age was 53 years (SD = 11.7, range = 23–74) and the majority (71%) lived with someone. Six of ten (63%) had experienced pain for 10 years or more. Musculoskeletal diseases were reported as the main reason for the pain (77%), and more than half of the participants (63%) had chronic conditions in addition to chronic pain, such as diabetes and chronic respiratory diseases. The baseline characteristics of the participants are presented in Table 1.

### Implementation of interventions

In total, six self-management course groups and six low-impact physical activity groups were delivered between September 2015 and December 2016 with 7–13 participants in each group. Ten participants (17%) did not attend the self-management courses, while 14 (23%) did not participate in the control group activities. Those allocated to the self-management course attended 4.2 of the six sessions on average, and 45 participants (75%) attended half or more of the sessions. The participants allocated to the low-impact physical activity groups attended, on average, 2.7 of the 6 sessions, and 32 participants (52%) attended half or more of the sessions.

In the intervention group, two adverse events were reported during the sessions; one participant had an anxiety attack, and one participant reported benign paroxysmal positional vertigo after performing one of the movement exercises. In the control group, there was one adverse event in which a participant strained a leg muscle during a walk. The symptoms of all of the reported events disappeared within a short time, and no adverse events were reported thereafter.

### Outcome

#### Primary outcome

The observed mean scores at 12 months for the primary outcome, PAM-13, were 66.7 for the intervention group and 62.2 for the control group (Table 2). The estimated mean difference between the groups was 4.0 (CI 95% -0.6 to 8.6, *p* = 0.085), which was was not statistically significant at the *p* ≤ 0.05 level.

**Table 2:** Observed mean (SD) at baseline, 6 months and 12 months. Estimated differences (95% Confidence Interval (CI)) within groups from baseline to 6 months and from baseline to 12 months, and differences between groups at 12 months

	Group	Observed			Estimated					
					Within groups Baseline to 6 months		Within groups Baseline to 12 months		Between groups 12 months	
		Baseline mean(SD)	6 months mean (SD)	12 months mean (SD)	Diff (95% CI)	*p*-value	Diff (95% CI)	*p*-value	Diff (95% CI)	*p*-value
PAM-13 (0–100) ↑	INTV	63.9 (13.2)	67.7 (14.5)	66.7 (14.3)	4.0 (0.4 to 7.5)	0.027	3.1 (−0.4 to 6.5)	0.084	4.0 (− 0.6 to 8.6)	0.085
	CTRL	63.0 (12.9)	64.9 (12.4)	62.2 (10.0)	1.4 (−2.2 to 5.0)	0.445	−1.0 (−4.5 to 2.6)	0.587		
BPI, severity (0–40) ↓	INTV	18.2 (6.5)	17.4 (7.4)	17.4 (6.8)	−0.8 (− 2.4 to 0.8)	0.320	− 0.5 (− 1.8 to 0.9)	0.486	0.6 (− 1.3 to 2.4)	0.526
	CTRL	18.8 (5.6)	18.1 (6.6)	17.4 (6.1)	−0.5 (−2.1 to 1.1)	0.550	−1.1 (−2.5 to 0.3)	0.124		
BPI, interference (0–70) ↓	INTV	29.2 (14.0)	27.9 (16.0)	25.9 (14.5)	−1.6 (−5.2 to 2.0)	0.379	−3.3 (−6.7 to 0.1)	0.061	−2.6 (−7.3 to 2.0)	0.269
	CTRL	32.6 (13.1)	30.8 (15.0)	31.0 (15.0)	−0.5 (−4.3 to 3.2)	0.779	−0.6 (− 4.2 to 2.9)	0.728		
BPI, pain relief (0–10) ↑(0-	INTV	3.4 (3.3)	4.0 (2.8)	6.3 (14.5)	0.6 (−0.2 to 1.4)	0.134	2.9 (0.5 to 5.2)	0.017	2.9 (−0.1 to 5.8)	0.055
	CTRL	3.5 (2.9)	3.4 (2.9)	3.4 (2.9)	−0.1 (−0.9 to 0.8)	0.894	−0.003 (−2.4 to 2.4)	0.998		
VAS, pain (0–100) ↓	INTV	62.7 (18.2)	53.9 (21.5)	55.5 (23.6)	−8.7 (−14.2 to −3.3)	0.002	−7.0 (− 12.5 to − 1.4)	0.014	−0.5 (− 7.6 to 6.7)	0.901
	CTRL	62.8 (15.1)	56.1 (19.3)	56.0 (19.6)	−6.5 (−12.1 to − 1.0)	0.021	−6.5 (− 12.2 to −0.8)	0.026		
HADS, depression (0–21) ↓	INTV	4.4 (3.0)	4.8 (3.9)	4.8 (3.5)	0.4 (−0.4 to 1.1)	0.329	0.3 (−0.5 to 1.0)	0.468	0.3 (−0.7 to 1.3)	0.525
	CTRL	5.1 (3.1)	5.1 (3.7)	5.0 (3.6)	0.2 (−0.6 to 1.0)	0.672	−0.1 (− 0.8 to 0.7)	0.883		
HADS, anxiety (0–21) ↓	INTV	7.8 (3.4)	7.1 (4.5)	7.2 (4.3)	−0.7 (−1.4 to 0.002)	0.051	−0.6 (− 1.3 to 0.2)	0.132	0.1 (− 0.9 to 1.2)	0.798
	CTRL	8.1 (3.6)	7.8 (3.7)	7.5 (3.1)	−0.5 (−1.3 to 0.2)	0.163	−0.7 (−1.5 to 0.1)	0.072		
PSEQ (0–60) ↑	INTV	38.1 (10.5)	39.1 (12.8)	39.1 (12.1)	1.0 (−1.5 to 3.3)	0.452	0.5 (−1.9 to 3.0)	0.678	1.5 (−1.9 to 4.9)	0.387
	CTRL	37.5 (10.4)	37.6 (10.8)	37.2 (11.2)	−0.7 (−3.2 to 1.7)	0.554	−1.0 (−3.5 to 1.6)	0.453		
SOC-13 (13–91) ↑	INTV	61.4 (12.4)	62.8 (15.2)	63.4 (15.5)	1.1 (−1.4 to 3.5)	0.401	1.1 (−1.4 to 3.5)	0.393	0.5 (−2.9 to 3.9)	0.771
	CTRL	61.8 (13.0)	63.7 (12.9)	62.1 (12.4)	1.5 (−1.0 to 4.0)	0.231	0.6 (−1.9 to 3.0)	0.657		
EQ-5D-5 L (0–1) ↑	INTV	0.63 (0.14)	0.64 (0.16)	0.63 (0.15)	0.02 (−0.01 to 0.05)	0.174	0.002 (− 0.03 to 0.03)	0.896	− 0.03 (− 0.1 to 0.01)	0.206
	CTRL	0.61 (0.14)	0.65 (0.14)	0.64 (0.16)	0.04 (0.004 to 0.07)	0.029	0.03 (−0.002 to 0.06)	0.069		
AIOS (0–100) ↑	INTV	46.3 (21.3)	51.8 (19.1)	45.1 (16.6)	6.5 (0.9 to 12.1)	0.023	0.2 (−5.8 to 6.1)	0.960	1.7 (−5.6 to 8.9)	0.649
	CTRL	43.4 (18.5)	45.5 (16.2)	43.3 (16.4)	0.8 (−5.0 to 6.5)	0.797	−1.5 (−7.7 to 4.6)	0.625		
Global health (1–5) ↑	INTV	2.1 (0.89)	3.5 (0.9)	3.6 (0.9)	1.4 (1.1 to 1.6)	< 0.001	1.4 (1.2 to 1.7)	< 0.001	−0.1 (−0.4 to 0.2)	0.363
	CTRL	2.2 (0.69)	3.6 (0.7)	3.8 (0.6)	1.4 (1.1 to 1.7)	< 0.001	1.6 (1.3 to 1.8)	< 0.001		
Physical activity (1–5) ↑	INTV	4.0 (0.87)	4.0 (1.0)	4.0 (0.9)	−0.002 (−0.2 to 0.2)	0.985	0.01 (−0.2 to 0.2)	0.927	0.01 (−0.3 to 0.3)	0.929
	CTRL	4.0 (1.02)	4.1 (0.8)	4.0 (0.8)	0.1 (−0.1 to 0.3)	0.356	−0.003 (− 0.2 to 0.2)	0.976		
30 s Chair to Stand ↑	INTV	12.5 (4.1)	13.3 (6.6)	15.0 (4.7)	0.9 (−0.4 to 2.2)	0.161	2.2 (1.4 to 3.1)	< 0.001	−0.5 (−1.7 to 0.7)	0.383
	CTRL	11.5 (4.0)	12.8 (5.6)	14.4 (3.7)	1.0 (−0.4 to 2.3)	0.153	2.8 (1.9 to 3.6)	< 0.001		

*SD*: Standard deviation; *INTV*: Intervention group; *CTRL*: Control group; *PAM-13*: Patient Activation Measure; *BPI*: Brief Pain Inventory; *VAS*: Visual analogue Scale; *HADS*: Hospital Anxiety and Depression Scale; *PSEQ*: Pain Self-Efficacy Questionnaire; *SOC-13*: Sense of Coherence; *EQ-5D-5 L*: EuroQoL 5 dimensions 5 level; *AIOS*: Arizona Integrative Outcomes Scale

Estimates presented are from linear mixed effects model (unadjusted) without random slope. ↑ Increase in scores indicates improvement

↓ Decrease in scores indicates improvement

The numbers of participants for each outcome at 6 months varied between 96 and 103 due to some missing responses

The numbers of participants for each outcome at 12 months varied between 85 and 100 due to some missing responses

There was a statistically significant change from the baseline to six months with improvement in patient activation for the intervention group (estimated mean change 4.0, CI 95% 0.4 to 7.5, *p* = 0.027) but not in the control group (estimated mean change 1.4, CI 95% -2.2 to 5.0, *p* = 0.445). After 12 months, there was no statistically significant change for PAM-13 within the groups, either for the intervention (estimated mean change 3.1, CI 95% -0.4 to 6.5, *p* = 0.081) or for the control group (estimated mean change − 1.0, CI 95% -4.5 to 2.5, *p* = 0.585).

#### Secondary outcomes

For the secondary outcomes, there were no statistically significant differences between the groups at the 12-month follow-up.

From the baseline to six-months, there were statistically significant changes within both groups with an improvement in the experience of pain during the previous week and on the global self-rated health measure. After 12 months, there were statistically significant changes within both groups with an improvement in the experience of pain during the previous week (intervention: -7.0, CI 95% -12.5 to − 1.4, *p* = 0.014; control: -6.5, CI 95% -12.2 to − 0.8, *p* = 0.026), for the global self-rated health measure (intervention: 1.4, CI 95% 1.2 to 1.7, *p* < 0.001; control: 1.6, CI 95% 1.3 to 1.8, *p* < 0.001) and for the 30-s Chair to Stand Test (intervention: 2.2, CI 95% 1.4 to 3.1, *p* < 0.001; control: 2.8, CI 95% 1.9 to 3.6, *p* < 0.001).

At the 12-month follow-up, there was no statistically significant differences between the groups in work status (no change 75% intervention group, 85% control group, *p* = 0.212) or in pain medication (no change 76% intervention group, 83% control group, *p* = 0.396). The differences in healthcare utilisation were only minimal when comparing the groups and no significant differences were found (*p*- values ranging from 0.272 to 0.558).

#### Post-hoc sub-group analysis

The exploratory post-hoc sub-group analysis showed that the mean change in PAM-13 from the baseline to twelve months increased for those with the two lowest levels of patient activation (level 1 and 2) (*n* = 28); 10.8 points for the intervention group and 9.2 points for the control group. There were only minor changes for those at patient activation level 3 (*n* = 61) (1.0 point for the intervention group and − 0.6 point for the control group). For those with the highest activation level at the baseline (level 4) (*n* = 30), there was a decrease with − 12.2 points for the control group and − 1.5 points for those in the intervention group. The test result for an overall interaction effect between the patient activation level at the baseline and allocation was not significant (*p* = 0.623).

## Discussion

After twelve months, there were no statistically significant differences between the intervention group and the control group either for the primary outcome patient activation, or for any of the secondary outcomes. Within both of the groups, there were statistically significant changes related to an improvement in pain experienced during the previous week, the self-rated health measure and the 30-s Chair to Stand Test from the baseline to the final follow-up.

As outlined, PAM-13 is a suitable primary outcome for measuring activation in self-management support interventions [83, 84]; however, at present, there is no consensus regarding a cut-off level to represent a meaningful change in the PAM-13. A study on patient education in a hospital setting in Norway, which showed a statistically significant improvement in PAM-13 on six points [47], informed the sample size calculation in the present study. Fowles and colleagues on the other hand suggested that a five-point difference in the PAM can be interpreted as a meaningful difference in PAM scores [85], whereas Turner and colleagues defined a meaningful improvement as four points on the PAM-13 scale [45]. Thus, the estimated mean difference of four points found in the current study is at best on the borderline of being a clinically relevant difference, although the finding was not statistically significant.

A possible reason for not finding an effect could be that the self-management skills and strategies introduced by the course did not result in changes that motivated the participants to include them as part of their pain management. Nicholas et al. [86] found that those who adhered regularly to self-management strategies presented by a multidisciplinary pain management programme (e.g., goal-setting, activity pacing, thought management and stretch exercises), had better outcomes one year later in comparison with those who adhered to them inconsistently or rarely during the programme. The attendance for the current self-management course was on average 4.2 of six sessions, and 75% of the participants attended half or more of the sessions. However, attendance is a poor proxy measure for adherence to behavioural changes [9] as the extent to which the participants practiced the strategies presented by the self-management course both during the course and after the course is unknown. Hence, the participants may not have applied the self-management strategies presented by the course, and if they did not, an effect could consequently be difficult to identify.

Furthermore, there were no organised follow-ups after the intervention, meaning that there was no additional support for the participants to maintain behavioural changes. New strategies should be practiced and result in positive experiences in order to change perceptions towards pain [19]. It is likely that those who experience not to succeed in managing their pain are those who reach out to health professionals for support, advice and guidance [9, 17]. It might be that the given intervention did not give sufficient support to prevail changes in how the participants perceived and acted upon their pain. Combining the self-management course with additional support in parallel with or over time, could possibly enhance the ability to self-manage [19] and to maintain newly learned behaviours [20], especially when considering that to self-manage chronic pain has been described as exhausting and as a constant struggle also after participating in interventions [19].

During the follow-up, there were improvements within each of the groups for the global self-rated health measure, the 30-s Chair to Stand Test and pain experienced during the previous week. As the intervention addressed persons with chronic pain, the changes in pain is of particular interest. The intervention group had an estimated change after 12 months from 62.7 to 55.5 in VAS pain indicating an improvement of approximately 11.5%, which is less than what has been considered minimal or little change when using VAS to measure pain (15–20%) [87]. No statistically significant improvements were found for pain severity and pain interference using the BPI. Thus, it is not likely that the intervention had a clinical meaningful impact on pain experience.

Given the variations in types of pain, diagnoses and associated challenges for those with chronic pain as well as the different environmental and treatment contexts, there is unlikely to be a single self-management method or strategy suitable for all [9]. To explore whether the course possibly could have had effect for some, an explorative post-hoc sub group analysis was done related to the participants PAM-level at baseline. In this, an improvement was observed for those at the lowest activation levels regardless of allocation, whereas those at the highest patient activation levels had a decrease in their PAM-13 score; however, more so in the control group than in the intervention group. As this was an explorative analysis and the test result for interaction between patient activation levels and allocations was not significant, no conclusions can be drawn. There are on the other hand RCTs which have shown that those at the lower patient activation levels have benefited the most from a web-based intervention for adults with chronic conditions [44] and self-referrals to a mental health treatment [43]. In contrast, a Finnish RCT on the effect of a patient portal with electronic messaging showed that those with the highest patient activation level at the baseline experienced the greatest effect from the intervention [88].

This diversity of effects indicates that it might be a connection between baseline patient activation-levels and the type of self-management intervention. One hypothesis could be that some interventions are better suited for those at certain activation levels. However, based on the description of the interventions in the studies referenced above [43, 44, 88], it is difficult to characterise the contents as advanced or not. Nevertheless, the observations of the current study along with findings from these studies provide intriguing considerations and raise interesting questions that should be investigated further. For instance, could the type of activities investigated in the current study be especially suited for the target group of the HLCs, meaning those who need help to change health behaviours and manage health challenges, and more specifically, those in the lower patient activation levels?

### Strength and limitations

The main strengths of this study is the RCT design and that the reporting of the study was done using guidance for reporting RCTs [89] and for complex interventions [90]. In addition, it is one of few studies to have evaluated the effect of a self-management intervention developed locally at an HLC.

The characteristics of the participants in the present study limits the generalisability of the findings. For example, people with chronic pain who experience major psychological or physical implications related to their pain condition, and thus are in need of more extensive interventions, were not included. More than 80% of the participants were women, which may also limit the generalisability of the findings. Previous population-based and epidemiological studies have indicated that more women than men report chronic pain [76, 91, 92] and other self-management studies have also reported samples with a majority of women [45, 93, 94], though not in the same proportion as in the current study. A possible explanation for few men within the study sample may be that men often find self-management support more appealing when it is perceived as action-oriented with a clear purpose offering personally meaningful information and practical strategies to integrate into daily life [95]. It is possible that the announcement of the intervention did not reflect this and that men did not respond for this reason.

It cannot be ruled out that other outcome measures would have been more sensitive to an effect from the intervention, but this is not very likely given the wide range of outcome measures covering domains recommended for chronic pain studies [87] and self-management [17, 24, 50]. However, some factors central to the participants might not have been covered, because in a qualitative study with a sample of the participants from this RCT it was found that hope and social support were central expectations towards participation in the interventions [82].

It should be mentioned that there are a number of contextual and procedural differences between the two trial activities, such as that the participants in the two groups received interventions of different lengths. This might have influenced the outcomes, in which it may be difficult to clarify what, other than the intervention itself, influenced the outcomes for the two groups. The number of participants who completed the questionnaires decreased gradually throughout the trial despite efforts to encourage participants to attend the follow-up appointments. Thus, the number of observations at follow-up was less than the sample size calculation and the power of the study was therefore lower than expected. Furthermore, for many of the outcomes measured in the study, the CIs were broad, and hence attached with low precision. This might be due to the heterogeneity of participants. The exploratory post-hoc sub-group analysis should ideally have been pre-planned. If the overall interaction between the sub-groups and allocations had been significant, the results would have been considered less reliable than those from the main analyses would.

## Conclusion

In this study, no long-term effect of the chronic pain self-management course was found compared with a low-impact physical activity intervention delivered via an easily accessible service on the primary outcome patient activation or on any secondary outcome. To understand more of how the intervention was perceived, the participants’ experiences related to the intervention should be investigated.

.

## Abbreviations

AIOS: Arizona Integrative Outcome Scale
BPI: Brief Pain Inventory
CBT: Cognitive Behavioural Therapy
CI: Confidence Interval
EQ-5D-5 L: EuroQoL 5 Dimensions 5 Level
HADS: Hospital Anxiety and Depression Scale
HLC: Health Life Centre
ICPC-2: International Classification of Primary Care 2. Edition
IMMPACT: Initiative on Methods, Measurement, and Pain Assessment in Clinical Trials
PAM-13: Patient Activation Measure
PSEQ: Pain Self-Efficacy Questionnaire
RCT: Randomised Controlled Trial
SD: Standard Deviation
SOC-13: Sense of Coherence
VAS: Visual analogue Scale
